# Conservation of Thermospermine Synthase Activity in Vascular and Non-vascular Plants

**DOI:** 10.3389/fpls.2019.00663

**Published:** 2019-06-11

**Authors:** Anna Solé-Gil, Jorge Hernández-García, María Pilar López-Gresa, Miguel A. Blázquez, Javier Agustí

**Affiliations:** Instituto de Biología Molecular y Celular de Plantas, Consejo Superior de Investigaciones Científicas – Universidad Politécnica de Valencia, Valencia, Spain

**Keywords:** plants, polyamines, thermospermine, evolution, development

## Abstract

In plants, the only confirmed function for thermospermine is regulating xylem cells maturation. However, genes putatively encoding thermospermine synthases have been identified in the genomes of both vascular and non-vascular plants. Here, we verify the activity of the thermospermine synthase genes and the presence of thermospermine in vascular and non-vascular land plants as well as in the aquatic plant *Chlamydomonas reinhardtii*. In addition, we provide information about differential content of thermospermine in diverse organs at different developmental stages in some vascular species that suggest that, although the major role of thermospermine in vascular plants is likely to be xylem development, other potential roles in development and/or responses to stress conditions could be associated to such polyamine. In summary, our results in vascular and non-vascular species indicate that the capacity to synthesize thermospermine is conserved throughout the entire plant kingdom.

## Introduction

Polyamines are positively charged aliphatic compounds with a widespread presence in all living organisms (Tabor and Tabor, 1984). The diamine putrescine (Put) and the triamine spermidine (Spd) are the most commonly found polyamines, while the tetra-amine spermine (Spm) is found in yeasts, most animals, seed plants and some bacteria (Pegg and Michael, 2010). Another tetra-amine, thermospermine (Tspm), has been detected in archaea, diatoms and plants, but not in animals or bacteria (Michael, 2016).

The ability to synthesize specific polyamines is clade-specific and is mainly the result of evolutionary adjustments in the polyamine biosynthesis pathway, reflected in the presence or absence of specific polyamine biosynthetic enzymes in given clades. Triamines and tetra-amines are mainly synthesized by a set of aminopropyl transferases, which are evolutionarily related to each other. It has been proposed that while Spm synthase (SPMS) genes in fungi and plants have likely emerged independently after duplication and neofunctionalization of Spd synthase (SPDS) genes (Minguet et al., 2008), the Tspm synthase (TSPMS) gene in plants was probably acquired through endosymbiosis of a cyanobacterium. However, it is arguable whether such gene originally encoded a TSPMS or an agmatine aminopropyl transferase (Pegg and Michael, 2010; Michael, 2016). All in all, the capacity to synthesize different polyamines in a given species is defined by (i) the presence or absence of specific polyamine biosynthetic enzymes and (ii) the degree of specificity of the polyamine biosynthesis enzymes toward their substrates. For instance, it has been reported that an aminopropyl transferase from the archaea *Pyrobaculum aerophilum* displays its highest specificity *in vitro* toward norspermidine, resulting in norspermidine biosynthesis, but it is also able to synthesize Tspm from Spd (Knott et al., 2007). Similarly, the gymnosperm *Pinus sylvestris* lacks a specific *SPMS* gene, but the aminopropyl transferase encoded by *PsSPDS* efficiently converts Put to Spd, as well as Spd to Spm (Vuosku et al., 2018). Both this flexibility in substrate recognition and the repeated independent generation of new aminopropyl transferases along evolution can be explained by the alteration of a few key residues in the active site of aminopropyl transferases that determine their characteristic substrate discrimination, as proposed through structural modeling and crystal structure comparisons of active sites (Wu et al., 2007; Minguet et al., 2008; Sekula and Dauter, 2018).

In plants, polyamines have been implicated in the response to stress and in the modulation of developmental processes (Chen et al., 2018). Correlations between specific endogenous levels of polyamines in plants under different stress conditions or during the progression of specific developmental processes have been extensively reported. In addition, the effects provoked by exogenous polyamines application have been largely documented. However, beyond such physiological reports, solid evidences for polyamines roles come from the analysis of loss of function mutants in polyamine metabolism genes. In this way, Put and Spd have been proved to be essential for life (Imai et al., 2004), while the tetra-amine Spm has been shown to be required for proper acclimation to salt, drought and heat stress (Yamaguchi et al., 2006, 2007; Sagor et al., 2013). Furthermore, both Spm and Tspm promote protection against bacterial pathogens (Gonzalez et al., 2011; Marina et al., 2013). However, the participation of Tspm could be a secondary effect of its primary function in vascular development (Vera-Sirera et al., 2010).

The most extensively studied role for a polyamine is that of Tspm in the regulation of xylem differentiation. The Arabidopsis *acaulis5* (*acl5*) mutant, impaired in TSPMS activity, displays stunted growth (Hanzawa et al., 1997, 2000) which has been associated to an increase in vascular cell proliferation, premature xylem cell death and miss-regulated lignin deposition (Clay and Nelson, 2005; Muniz et al., 2008). In Arabidopsis, the *ACL5* gene is specifically expressed in developing xylem cells (Clay and Nelson, 2005; Muniz et al., 2008), and the HD-ZIPIII transcription factor AtHB8 -which directs xylem differentiation and displays the same expression domain as *ACL5*- mediates its induction by auxin (Baima et al., 2014). Auxin has been shown to promote cell proliferation by ensuring the formation of complexes between Target of Monopteros5 (TMO5) and Lonesome Highway (LHW), leading to the induction of cytokinin biosynthesis (De Rybel et al., 2013, 2014). Recent evidence suggests that Tspm is part of a negative feed-forward loop triggered by auxin that maintains proliferation of vascular cells within the correct range (Katayama et al., 2015; Vera-Sirera et al., 2015). The mechanism involves the promotion of translation of a small family of Suppressor of ACL5 (SACL) transcriptional regulators (Imai et al., 2006; Kakehi et al., 2008) which compete with TMO5 for the formation of inactive SACL-LHW complexes (Katayama et al., 2015; Vera-Sirera et al., 2015). This is not the only case in which polyamines have been implicated in translational regulation (Hanfrey et al., 2002, 2005).

Systematic sequencing of plant transcriptomes and genomes has revealed the presence of *ACL5* putative homologs in all plant lineages of vascular and non-vascular plants (including algae), raising the following questions: Do all putative *ACL5* genes across plant lineages encode enzymes with *bona-fide* TSPMS activity, even those present in non-vascular species? Is the expression of these genes associated to xylem development in all vascular plants? To start answering these questions, we have (i) examined the enzymatic activity of the ACL5 homologs of several species in key plant lineages, (ii) searched for Tspm presence in vascular and non-vascular plants and (iii) studied the *ACL5* expression pattern in vegetative and reproductive organs of several seed-plant species at different developmental stages.

## Materials and Methods

### Plant Material and Growth Conditions

*Arabidopsis thaliana* Columbia-0 (Col-0) plants were grown on MS media with sucrose (1%) under long-day conditions (16 h light, 8 h dark) at 22°C in a growth room. Samples for polyamine analysis and RNA extraction from Col-0 were taken after 15 days of growth. *P. abies* plant material was collected from adult trees that were identified in Catalonia (Spain) and frozen at –80°C until further analysis. *S. lepidophylla* plants were obtained from an external vendor, and hydrated before freezing the material at –80°C for further analysis. *P. patens* plant material was obtained from Jesús Vicente Carbajosa’s Lab (CBGP-Madrid, Spain) and grown in BCD medium + 1 mM Ca^2+^ under continuous light conditions in a growth room (Ashton and Cove, 1977). *M. polymorpha* Tak-1 plants were grown on ½ strength Gamborg’s B5 medium for 15 days before tissue recollection under continuous light conditions in a growth room. *C. reinhardttii* plant material was obtained from Federico Valverde’s Lab (IBVF-Sevilla, Spain).

### Phylogenetic Analysis

Sequences used in this study were obtained by extensive BLAST analysis (tblastn) using the Arabidopsis thermospermine synthase (ACL5-AT5G19530), spermine synthase (SPMS-AT5G53120), and spermidine synthase 1 and 2 (SPDS1-AT1G23820, SPDS2-AT1G70310) genes as baits in the NCBI^1^, Phytozome^2^ and OneKP^3^ databases. All the sequences were managed using the Benchling tool^4^. Alignment of the sequences was done with using the MUSCLE algorithm (Siebers et al., 2017) included in the SeaView 4.6.4 GUI (Gouy et al., 2010), with 16 iterations, default clustering methods, gap open score of –2.7, and hydrophobicity multiplier of 1.2, followed by manual curation. To select the best-fit model of amino acid substitution, the ProtTest v3.4.2 (Darriba et al., 2011) was used on final multiple sequence alignment, together with AIC model for ranking. Maximum likelihood tree was produced with PhyML v3.1 (Guindon et al., 2010), using the best-scored model of amino acid substitution. Statistical significance was evaluated by bootstrap analysis of 1000 replicates. Phylogenetic tree graphical representations were initially generated using FigTree (version 1.4.3) software^5^, and final cartoons edited manually.

### RNA Extraction and PCR Analysis

Total RNA was isolated from 200 mg of frozen powdered tissues using RNeasy plant mini kit (Qiagen) for *A. thaliana* and *M. polymorpha* tissues following the manufacturer’s instructions. RNA from *C. reinhardtii*, *P. patens*, *S. lepidophylla* and *P. abies* was extracted by Trizol-Chloroform method as described (Siebers et al., 2017). cDNA synthesis was performed on 1 μg DNase-treated RNA using PrimeScript^TM^ 1st strand cDNA synthesis kit (Takara). The resulting cDNA was used for PCR analysis with target gene-specific primers (Supplementary Table S2).

### Cloning and Expression in Yeast

Coding sequence regions of thermospermine synthase genes from the selected species were obtained either from cDNA (*M. polymorpha*, *S. lepidophylla* and *A. thaliana*) or synthesized (Integrated DNA Technologies, IDT). The primers used for amplification are summarized in Supplementary Table S2. For plasmid construction, the CDS of the genes was cloned into pDONR207 through Gateway recombination (Invitrogen) and eventually into the destination vector pAG426GPD-ccdb-HA (a gift from Susan Lindquist; Addgene plasmid number 14252) for expression in yeast. The *A. thaliana* thermospermine synthase CDS was cloned into pCR8 through Golden Braid technology (Sarrion-Perdigones et al., 2011) and then shifted to Gateway technology to clone the CDS into the pDEST like the other species.

Yeast strain BY4741 (*MAT*a *his3 leu2 met15 ura3*), kindly provided by Ramón Serrano’s lab (IBMCP-Valencia, Spain), was grown on Synthetic Defined (SD) medium and transformed with the pAG426GPD containing the CDS of interest by LiAc/ssDNA/PEG. Selected transformants were grown on 50 mL liquid SD medium lacking Trp and Ura for 2 days, then harvested by centrifugation (about 200 mg of pellet) and frozen for further polyamine quantification.

### Polyamine Quantification

Polyamine extraction from tissues and standards was done with a modification of a previously described method (Naka et al., 2010) as follows. About 1 g of plant samples in 3 replicates were frozen in liquid nitrogen and kept at –80°C until use. The tissue was ground with mortar and pestle (in the case of yeast pellets, the cells were broken with glass balls) and resuspended in 2.5 mL of 5% perchloric acid (PCA) in a 15-mL tube. At this point 500 μL of the internal standard (diethylamine 1 mM, DEA) was added to the 5% PCA. The samples were kept on ice for 1 h and centrifuged at 4°C for 20 min at 15000×*g*. The whole supernatant was collected (between 2 and 3.5 mL) and transferred to a fresh 15 mL falcon tube. For each mL of supernatant, 0.66 mL of 2 M NaOH was added. Then, benzoylation started with the addition of 10 μL of benzoyl chloride. After 1 min vortex, plant extracts were left at room temperature for 20 min. Then, for each mL of initial supernatant used, 1.33 mL of saturated NaCl solution was added. Two mL of diethyl ether was added, vortexed, and centrifuged at 3000*×g* for 1 min for the separation of the phases. The supernatant was transferred to a new pyrex vial and dried completely using N_2_. The remaining polyamines were resuspended in 130 μL methanol and filtered with a filter syringe (pore size 0.2 μm). Then, the filtrate was transferred to a plastic vial for HPLC analysis. Briefly, 30-μL aliquots were injected through a Waters 717plus autosampler into a 1525 Waters Binary HPLC pump equipped with a 996 Waters PDA detector and using a Luna C18(2) (Phenomenex) column (250 × 4.6 mm, i.d. 5 μm). The column was equilibrated with 58% solvent A (acidic H_2_O containing 10 mL acetic acid for each liter of distilled water) and 42% solvent B (acetonitrile). Elution was carried out at room temperature and for polyamine separation a 1 mL min^–1^ flow rate was used the isocratic gradient of 42% acetonitrile for 25 min. Then, the column was washed with 42–100% acetonitrile within 3 min and kept at 100% acetonitrile for 10 min. Eventually, the column was equilibrated with 42% acetonitrile for 17 min before the next injection. Detection of polyamines was performed at 254 nm.

### Expression Analysis

Data for *TSPMS* gene expression in *A. thaliana, P. trichocarpa, M. truncatula, S. lycopersicum, Symphytum tuberosum, O. sativa, G. max, V. vinifera, B. distachyon, E. grandis, P. abies, Ananas comosus* and *P. patens* in different tissues was gathered from the Bio-Analytic Resource for Plant Biology (BAR^6^). Expression data was classified according to the organ tissue (stem, root, flower and leaf) and tissue age (mature or young). The total expression data per gene and species in the target organs was used to calculate the percentage of expression of the *TSPMS* gene in each organ. Heatmaps were drawn using the Matrix2png tool^7^.

## Results

### Identification of Thermospermine Synthase Genes Across Plant Lineages

**FIGURE 1:**
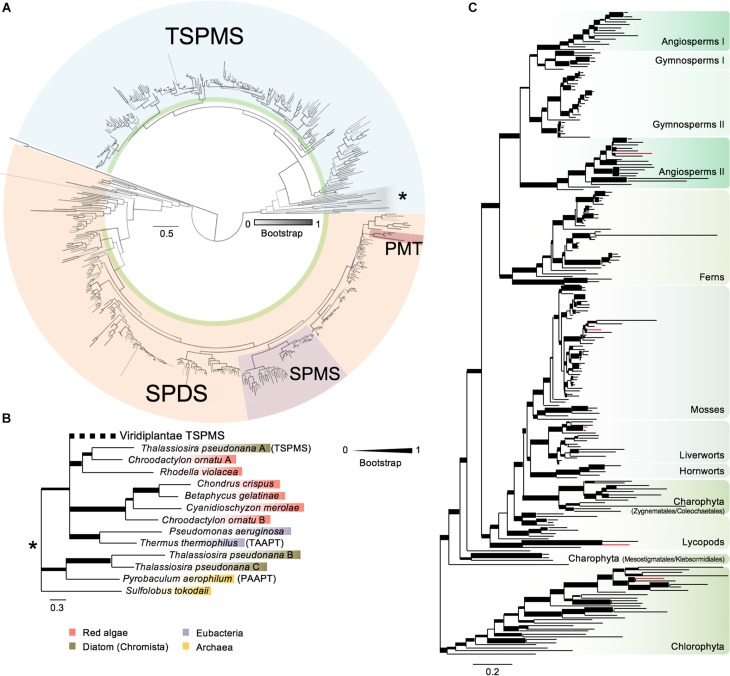
Phylogenetic analysis of polyamine aminopropyl transferases. **(A)** Phylogenetic analysis of polyamine aminopropyl transferases protein sequences with special focus in plant sequences. Support values associated with branches are maximum likelihood bootstrap values from 1000 replicates depicted as a color range (gray scale). Black branches indicate a bootstrap of 1 (100% support). Blue background indicates TSPMS clade sequences and light brown SPDS-related sequences. The remaining sequences belong to metazoan SPMS used as outgroups to root the tree. Purple and dark brown indicate SPMS sequences from Spermatophyta and PMT sequences from Solanaceae/Convolvulaceae, respectively. Green ribbons mark Viridiplantae sequences. Gray shaded sequences with an asterisk indicate basal branches of the TSPMS clade. **(B)** Phylogenetic tree of TSPMS basal branches extracted from panel **(A)**, marked with an asterisk. Support values associated with branches and displayed as bar thickness are maximum likelihood bootstrap values from 1000 replicates. Values under 0.75 have been merged to ease visualization. Names in brackets indicate experimentally tested forms of aminopropyl transferases: TSPMS, thermospermine synthase; TAAPT, triamine/agmatine aminopropyl transferase; PAAPT, polyamine aminopropyl transferase. Viridiplantae sequences have been collapsed. **(C)** Phylogenetic analysis of Viridiplantae TSPMS from tree **(A)**. Support values associated with branches and displayed as bar thickness are maximum likelihood bootstrap values from 1000 replicates. Background colors indicate sequences from major lineages of plants. Red colored branches indicate chosen sequences for experimental analysis. Scale bars in panels **(A–C)** indicate substitution per residue distances.

Polyamine biosynthesis enzymes display high degree of similarity (Hashimoto et al., 1998; Panicot et al., 2002; Teuber et al., 2007). Therefore, to guarantee accurate identification of putative *ACL5* orthologs, we screened the Phytozome and OneKP databases using as baits the Arabidopsis *ACL5* sequence, and also the sequences of the two Arabidopsis genes encoding SPDS, and the single gene for SPMS (see Section “Materials and Methods” for details). We obtained 542 sequences covering every major plant lineages and included members of representative bacteria, archaea and other non-plant eukaryotic groups, which were aligned and used for the construction of a phylogenetic tree (Figure 1A and Supplementary Files S1–S3). This is the most extensive phylogenetic study of plant polyamine aminopropyl transferases done to date, and it not only confirms some of the previous evolutionary models (Hashimoto et al., 1998; Panicot et al., 2002; Teuber et al., 2007; Minguet et al., 2008), but also provides new details on certain key events.

First, our analysis expands the presence of *ACL5* homologs and *SPDS* genes to all plant lineages not previously studied, like charophytes and chlorophytes (Figure 1A). Second, the exclusive origin of *SPMS* genes in angiosperms, previously proposed based on only a few sequences (Minguet et al., 2008), is now set in the common ancestor of all Spermatophytes with more sequences from gymnosperms and their absence in multiple genomes of other land plants. Third, we find indications for a possible transfer of an additional SPDS of Holomycota origin to Setaphyta, based on the presence of extra copies in the genomes of the bryophyte *Sphagnum* and of several liverworts that unequivocally cluster with some sequences of that class (Supplementary Files S1–S3). Fourth, as proposed in previous analyses (Minguet et al., 2008; Takano et al., 2012), our more extensive search still identifies *ACL5* orthologs only in plants, red algae, diatoms, archaea and bacteria (Figures 1A,B). The new data are compatible with the proposed model that TSPMS activity in Archaeplastida has a prokaryotic origin, while the common root with the Archaea *Sulfolobus* and *Pyrobaculum*, of two additional copies of *ACL5* orthologs in *Thalassiosira pseudonana* suggests a possible second event of horizontal gene transfer to Chromista (Figure 1B). And fourth, there are indications of an early *ACL5* duplication in Spermatophyta, followed by several species-specific losses of one of the copies within the angiosperms (some Brassicaceae like *Arabidopsis thaliana*, some Poaceae) (Figure 1C).

### Thermospermine Is Synthesized Across All Plant Lineages

**FIGURE 2:**
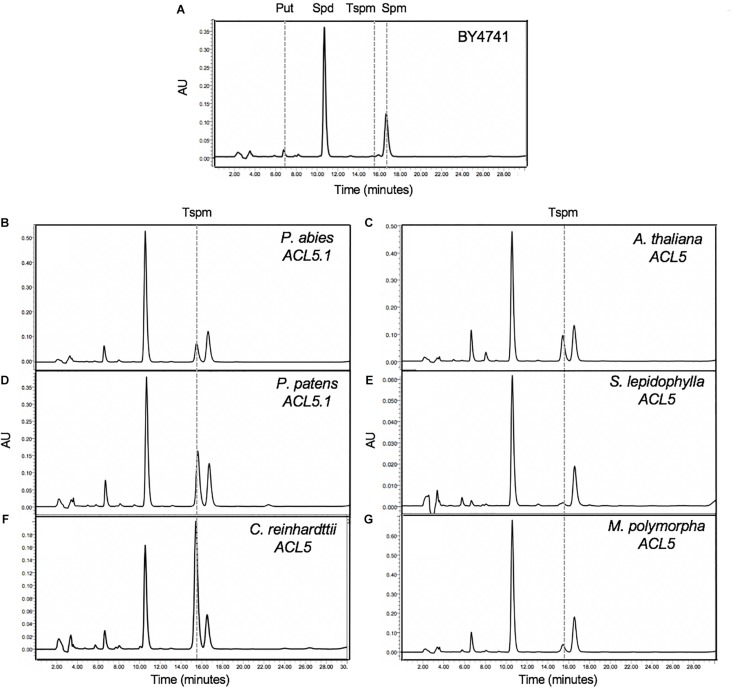
Functional analysis of *ACL5* orthologs. *Saccharomyces cerevisiae* BY4741 strain, unable to produce Tspm **(A)**, was transformed with the putative ACL5 from *P. abies* **(B)**, *A. thaliana* **(C)**, *P. patens* **(D)**, *S. lepidophylla* ACL5.1 **(E)**, *C. reinhardtii* **(F)** and *M. polymorpha* **(G)**, and the ability to produce Tspm was tested with an HPLC analysis. Graphs show the polyamine profile of yeast extracts after benzoylation and fluorescence detection with the help of a fluorimeter. Tspm, thermospermine; AU, absorbance units.

Our phylogenetic analyses identified at least one putative *ACL5* ortholog in all studied non-vascular plant species (Figure 1C). Since the only confirmed function for Tspm in plants is the regulation of xylem maturation dynamics (which, by definition, does not occur in non-vascular plants), we wondered whether the identified sequences encode enzymes displaying actual TSPMS activity. Therefore, we expressed the *ACL5* homologs of representative plant lineages in yeast, an organism that is unable to produce Tspm. Among the non-vascular plants, we tested *ACL5* homologs of a chlorophyte (*Chlamydomonas reinhardtii*), a liverwort (*Marchantia polymorpha*) and a moss (*Physcomitrella patens* (Supplementary Table S1). As representative vascular plants we chose one lycophyte (*Selaginella lepidophylla*), and one gymnosperm (*Picea abies*), with the angiosperm *A. thaliana* as a control (Supplementary Table S1). As previously shown, HPLC analysis showed the presence of Put, Spd and Spm, but not Tspm, in the extracts of a wild-type yeast strain transformed with an empty plasmid (Figure 2A). In contrast, all the tested ACL5 homologs allowed the production of Tspm in yeast (Figures 2B–G) demonstrating that these genes encode enzymes with TSPMS activity. This is in accordance with the previously reported partial complementation of the Arabidopsis *acl5* mutant by one of the *PpACL5* orthologs (Takahashi and Kakehi, 2010). The observed differences in Tspm production between the different *ACL5* genes suggest either species-specific variation in TSPMS activity kinetic parameters or in the variable capacity of yeast to express the different heterologous genes.

**FIGURE 3:**
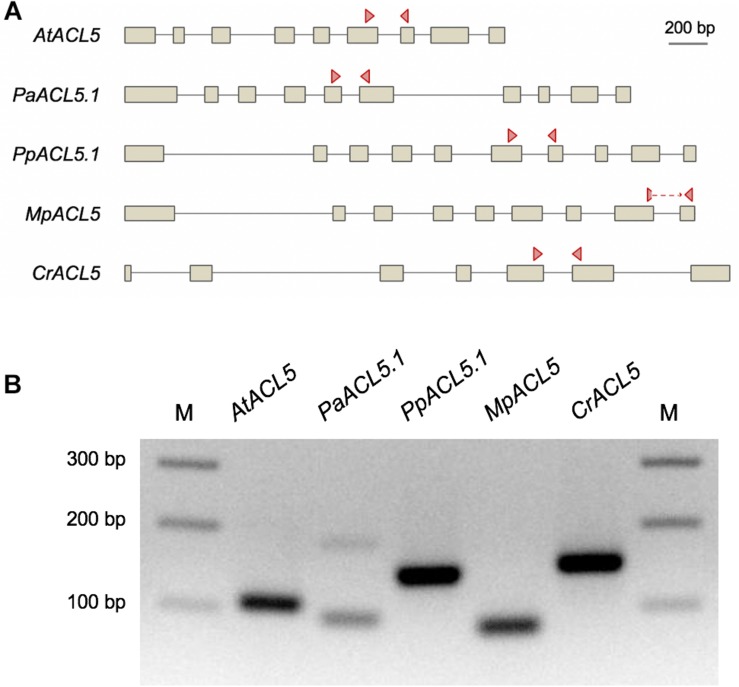
Detection of the expression of *ACL5* homologs in several plant species by PCR. **(A)** Schemes of the genes selected for this analysis. Exons are represented as boxes, and arrowheads indicate the primer pairs used for PCR detection, which are located on different exons, except for MpACL5, in which one of them spans an exon-exon junction. **(B)** PCR products amplified from cDNA. Plant material for RNA extraction and later cDNA synthesis was grown in the same standard conditions than the plants used for polyamine quantification by HPLC analysis.

To check whether the presence of ACL5 orthologs with TSPMS activity correlated with the ability of these species to synthesize Tspm *in vivo*, we examined the polyamine levels in samples of these plants grown in standard conditions (see Section “Materials and Methods”). As expected, all vascular and non-vascular species accumulated Put and Spd to different levels (Table 1). For instance, Put levels in the chlorophyte *C. reinhardtii* were between one and three orders of magnitude higher than in the land plants examined, while Spd levels were generally higher in the land plants than in *C. reinhardtii*, except for *P. patens*. On the other hand, Spm was detectable in *A. thaliana* and *P. abies*, in agreement with previous reports for the occurrence of this tetraamine in seed plants (Gonzalez et al., 2011; Vuosku et al., 2018) and the presence of SPMS orthologs in this clade (Figure 1A). The detection of Spm in *S. lepidophylla* despite the absence of SPMS orthologs in lycophytes indicates that this tetra-amine might be synthesized by a less strict SPDS, as already suggested for *P. sylvestris* (Vuosku et al., 2018). With respect to Tspm, all the species tested were able to synthesize this polyamine at different levels, varying between 1.13 nM/g (fresh weight) in *C. reinhardtii* and 154 nM/g in *P. abies*. It is worth noting that, while previous reports show that Tspm tends to accumulate less than Spm in Arabidopsis (Naka et al., 2010; Rambla et al., 2010; Cai et al., 2016; Yoshimoto et al., 2016), in the species, tissues and conditions analyzed here, the two polyamines showed comparable levels. Although this result does not demonstrate that the *ACL5* orthologs identified in all the selected species are responsible for the Tspm synthesis shown here, this possibility is further supported by the observation that these genes are actually expressed in the same growth conditions as the ones used for Tspm quantification (Figure 3).

**TABLE 1:** Polyamine quantification in plant tissues.

	**Polyamine concentration (nmol/g FW ± SE)**			
	**Putrescine**	**Spermidine**	**Spermine**	**Thermospermine**
*A. thaliana*	10.76 ± 3.19	52.34 ± 15.48	2.14 ± 0.35	4.69 ± 1.77
*P. abies*	37.74 ± 5.31	131.09 ± 20.99	42.84 ± 12.39	154.06 ± 19.52
*S. lepidophylla*	77.59 ± 12.17	123.59 ± 24.80	1.21 ± 0.27	17.27 ± 3.75
*P. patens*	2.58 ± 0.54	19.87 ± 2.42	n.d.	10.7 ± 3.01
*M. polymorpha*	15.26 ± 5.06	53.88 ± 13.32	n.d.	1.37 ± 0.19
*C. reinhardttii*	1032.51 ± 16.62	27.44 ± 1.18	n.d.	1.13 ± 0.15

*Polyamine quantification from *A. thaliana, P. abies, S. lepidophylla, P. patens, M. polymorpha*, and *C. reinhardttii* tissues. Polyamines were extracted and benzoylated from fresh tissues and measured with an HPLC analysis. Polyamine measurements were done in triplicates. FW, Fresh Weight; SE, Standard Error; n.d., not detected.*

### Differential *ACL5* Expression Dynamics in Vegetative and Reproductive Organs Throughout Development in Seed Plants

Tspm synthase activity has been associated to xylem development in *A. thaliana* (Muniz et al., 2008; Vera-Sirera et al., 2015), *Populus trichocarpa* (Milhinhos et al., 2013) and *P. sylvestris* (Vuosku et al., 2018). To investigate whether this association can be extended to other vascular plants and to point to additional potential roles in these species, we took advantage of the vast amount of publicly available transcriptomic data and examined the expression pattern of the *ACL5* orthologs in vegetative and reproductive organs of several vascular species at different developmental stages.

**FIGURE 4:**
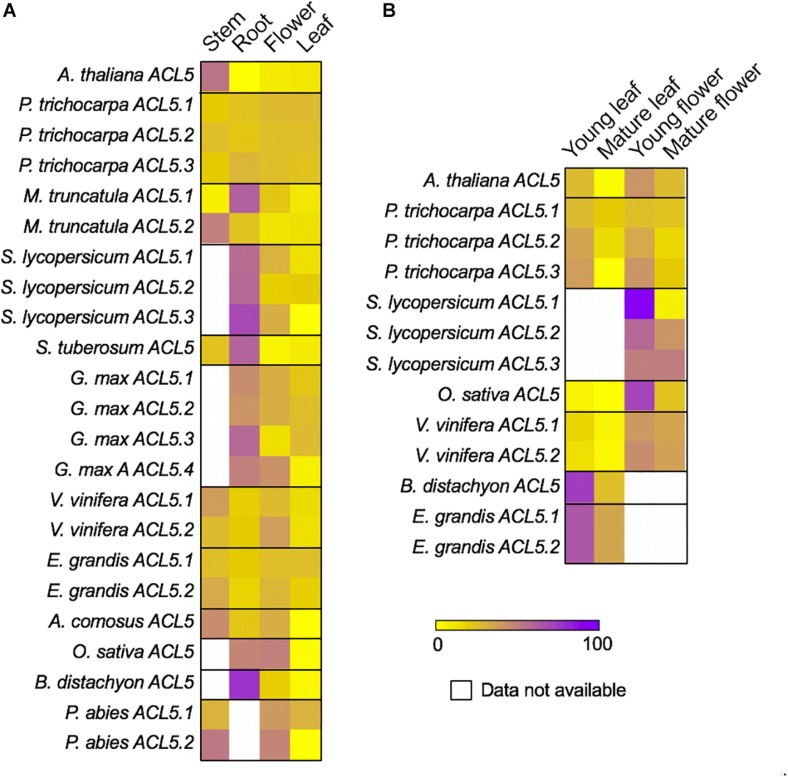
Differential expression of TSPMS genes in representative seed plants. Relative expression data of TSPMS is shown as percentage of expression of each gene per species. **(A)** Expression data of TSPMS in *A. thaliana, P. trichocarpa, M. truncatula, S. lycopersicum, S. tuberosum, G. max, V. vinifera, E. grandis, A. comosus, O. sativa, B. distachyon* and *P. abies* in stem, root, flower and leaf. **(B)** Expression data of TSPMS in from *A. thaliana, P. trichocarpa, S. lycopersicum, O. sativa, V. vinifera, B. distachyon* and *E. grandis* in different developmental stages of leaf and flower.

Although for some species we did not find data for stem or root, the available transcriptomic data shows that, for all studied species, *ACL5* transcripts tend to accumulate more in vegetative than in reproductive organs (Figure 4A). In the species for which transcriptomic data exists for both stem and root, *ACL5* expression is mostly higher in stems than in roots (7 out of 13 of the ACL5 homologs), while only in two cases is the opposite behavior observed. In *Medicago truncatula*, where two *ACL5* paralogs exist, we found that one of them is more prominently accumulated in the stem, while the other one accumulates more in the root. Since our analyses are based on percentage of transcript accumulation across organs, in the species for which no transcriptomic data is available for stem tissue (i.e., *Solanum lycopersicum*), the proportion of transcript accumulation appears to be highly enriched in roots. For the same reason, in general, we regard the cases in which we found strong *ACL5* accumulation in flowers to the non-availability of expression data for stem or root (i.e., *Oryza sativa* or *Glycine max ACL5#4*). Only in the case of *Vitis vinifera* (where transcriptomic data was available for all tested organs) we found that one of the paralogs showed a (slight) preferential expression in flowers than in vegetative organs. In *P. trichocarpa*, where three paralogs exist, we found that none of them display preferential expression for any of the analyzed organs.

We also observed a marked preference of *ACL5* expression for young vs. mature organs (Figure 4B). In *A. thaliana, P. trichocarpa, O. sativa* and *V. vinifera*, this was visible in both flowers and leaves. There was no preferential expression between leaves and flowers except for the case of *V. vinifera*, in which both paralogs displayed higher preference for flowers than for leaves. For *S. lycopersicum*, we only found transcriptomes for young and mature flowers, while in *Brachypodium distachyon* and *Eucalyptus grandis*, we only found transcriptomes for young and mature leaves, and in all of them we found enriched *ACL5* expression in young organs. These results indicate a fairly widespread expression of *ACL5* homologs across organs, and mostly in young tissues with active vasculature development.

## Discussion

Our current knowledge about thermospermine is that its only confirmed function in plants is the coordination of xylem maturation. However, previous phylogenetic work reported the existence in the genomes of non-vascular plants of sequences clustering with TSPMS (Minguet et al., 2008; Pegg and Michael, 2010), implying that Tspm might exist and, perhaps, play developmental and/or stress response-related roles also in such species. On one hand, as mentioned above, sequence similarity between all polyamine biosynthesis enzymes – especially those using putrescine or spermidine as substrates- has been extensively reported (Hashimoto et al., 1998; Panicot et al., 2002; Teuber et al., 2007). On the other hand, the sequencing of large numbers of genomes and transcriptomes of non-vascular plant species is relatively recent, and therefore previously published comparative analyses missed key clades in plant phylogeny.

**FIGURE 5:**
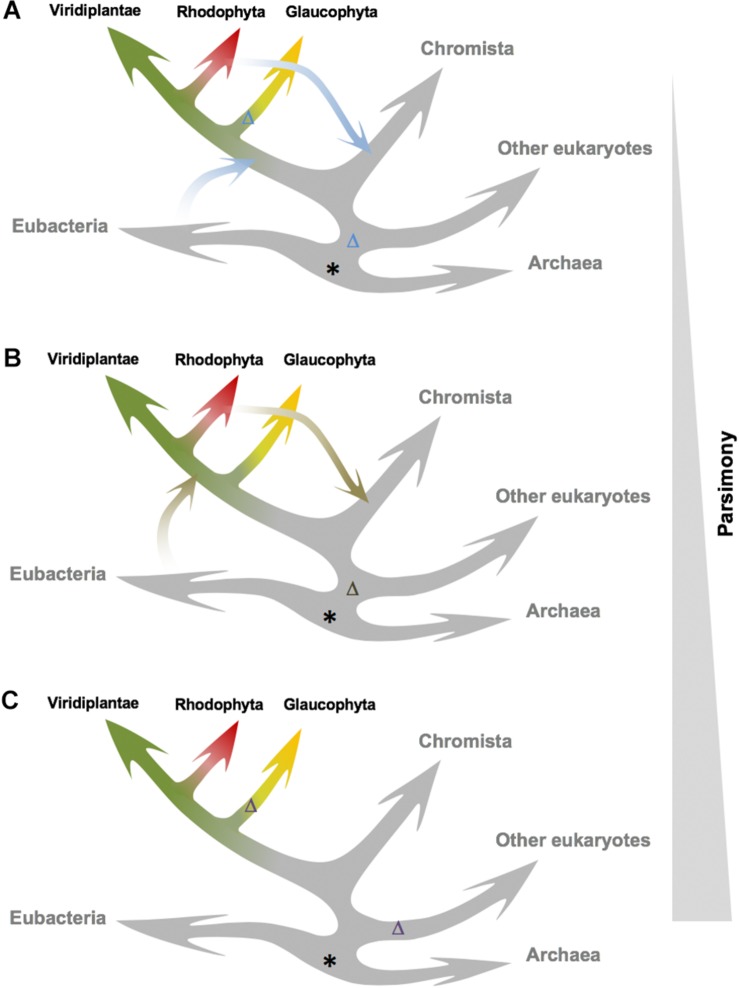
Alternative models for TSPMS activity origin and evolution in plants. The three models are ordered by increasing parsimony. **(A)** The ancestor of TSPMS activity in LUCA (possibly with a different activity) is lost in LECA; then it is acquired by Archaeplastida via HGT, and lost again in Glaucophyta. Chromista acquire it through secondary endosymbiosis. **(B)** Similar to the previous one, but Archaeplastida acquire the ACL5 ortholog after divergence of Glaucophyta. **(C)** The ancestor of TSPMS activity in LUCA is lost early in major eukaryotic clades, after the divergence of the green lineage, followed by an additional loss in Glaucophyta. Putative origin of TSPMS genes is marked with an asterisk, and predicted loss of TSPMS genes is marked with a Δ.

It has been proposed that TSPMS had appeared in plants by endosymbiosis of a cyanobacterium (Minguet et al., 2008). Our data are compatible with three alternative hypotheses in this respect (Figure 5). The most parsimonious one is that TSPMS was already present before Eubacteria and Archaea and was lost in the major eukaryotic branch (Figure 5C). A second loss would have occurred during the divergence of Glaucophyta, explaining the absence in such branch and the presence in the Chromista, Rhodophyta and Viridiplantae branches. A less parsimonious, but plausible possibility is that TSPMS was lost in the last eukaryotic common ancestor (LECA) and that it then was transferred either from Eubacteria or from Archaea (i) to Archaeplastida after Glaucophyta had diverged and (ii) to Chromista (Figure 5B). A third, less parsimonious hypothesis would postulate that TSPMS was lost in LECA and transferred either from Eubacteria or from Archaea to the early lineage of Archaeplastida and to Chromista (Figure 5A). TSPMS would have been lost again in Glaucophyta. However, phylogenetic positioning of TSPMS points to the horizontal transfer of TSPMS genes from Rhodophyta to diatoms (and possibly other algal groups originated during secondary chloroplast acquisition), thus excluding this latter hypothesis, which is also based in a very low sampling among Chromista representatives. Given that it is almost practically impossible to differentiate between the two remaining models, the hypothesis that contemplates only one loss and two reported horizontal transfers seems the most likely one (Figure 5B).

The identification of TSPMS activity in non-vascular plants clearly argues for possible functions of Tspm other than in xylem development. It has been proposed that Tspm might play a role in defense against stress conditions (Gonzalez et al., 2011; Marina et al., 2013), so it could also be the case that such activity is conserved across vascular and non-vascular plants and that developmental functions – either related with vascular development or with other, yet to be identified, processes – were only acquired in vascular plant lineages. In any case, further experimentation using mutant, overexpression and marker lines in emerging non-vascular model plant species such as *M. polymorpha* or *P. patens* might provide further information about what developmental and/or stress related processes might be controlled by this polyamine.

The *in silico* analyses of relative abundance of *ACL5* transcripts in different organs in representative seed-plant species presented here aimed at carrying out a first approach toward understanding whether, apart from coordinating xylem maturation, TSPM plays any other role in vascular plants. The higher prevalence of *ACL5* transcripts in vegetative than in reproductive organs and in young (presumably developing) than in mature organs argues for higher association of *ACL5* expression in organs developing more proportion of vascular tissues (vegetative) at early developmental stages, when vasculature is probably developing. However, the presence of *ACL5* transcripts in reproductive organs and in mature vegetative organs also argues for potential other (maybe less prominent) activities of TSPM in plants.

In summary, our work shows that Tspm exists in non-vascular plants and that there is a correlation between Tspm presence, TSPMS activity and *ACL5* expression throughout vascular and non-vascular plant lineages. Our results indicate that Tspm might play developmental and/or stress-related roles (apart from the described role in xylem development) not only in non-vascular but also in vascular plants. Future experimentation will shed new light on such intriguing topic.

## Data Availability

All datasets generated for this study are included in the manuscript and/or the Supplementary Files.

## Author Contributions

AS-G, JH-G, MB, and JA conceived and designed the work. AS-G, JH-G, and ML-G performed all *in silico* and experimental analyses. JA and MB wrote the first draft of the manuscript, to which all authors contributed.

## Conflict of Interest Statement

The authors declare that the research was conducted in the absence of any commercial or financial relationships that could be construed as a potential conflict of interest.

## Footnotes

1 http://www.ncbi.nlm.nih.gov

2 http://www.phytozome.net

3 http://db.cngb.org/onekp/

4 https://benchling.com

5 http://tree.bio.ed.ac.uk/software/figtree/

6 http://bar.toronto.ca

7 https://matrix2png.msl.ubc.ca/
