# Supplementary material for: Conservation of Thermospermine Synthase Activity in Vascular and Non-vascular Plants
Source: Front Plant Sci. 2019 Jun 11;10:663. doi: 10.3389/fpls.2019.00663 (PMC6579911; doi:10.3389/fpls.2019.00663)
Supplement: Supplementary file 4 [file Data_Sheet_4.PDF]

**Table S1.** ***ACL5*** **orthologs in the selected species**

| Clade | Species | Gene | Code |
|---|---|---|---|
| Angiosperms | *Arabidopsis thaliana* | *AtACL5* | AT5G19530.1 |
| Gymnosperms | *Picea abies* | *PaACL5.1* | PAB00026429 |
| | | *PaACL5.2* | PAB00036924 |
| Lycophytes | *Selaginella lepidophylla* | *SlACL5.1* | onekp:ABIJ_scaffold_2009837 |
| | | *SlACL5.2* | onekp:ABIJ_scaffold_2009492 |
| Bryophytes | *Physcomitrella patens* | *PpACL5.1* | Pp3c5_6270V3 |
| | | *PpACL5.2* | Pp3c6_27380V3 |
| | | *PpACL5.3* | Pp3c27_3260V3 |
| Liverworts | *Marchantia polymorpha* | *MpACL5* | Mapoly0012s0100 |
| Green algae | *Chlamydomonas reinhardtii* | *CrACL5* | Cre06.g251500 |

**Table S2. Oligos used for PCR amplification**

| Gene | Forward | Reverse | cDNA product (bp) | gDNA product (bp) |
|---|---|---|---|---|
| ***AtACL5*** | AACATTCTCAAACCCAAGCTTAGC | ATGGTGTTGTAGATTGATGTGAAGACT | 107 | 213 |
| ***PaACL5.1*** | CCGCCTTGAGCTCGTTATCAAC | CTGGGTCTGCAAGGTCTCCTAC | 92 | 160 |
| ***PpACL5.1*** | CAGTGGCAGGAGGTCCTTGTTA | GGAGTAGACCTCTGTGTGCGTT | 140 | 277 |
| ***MpACL5*** | GTGAGAAAATCGTTGGCCCAGG | TTCGCATTGGTTCCATGTCC | 83 | – |
| ***CrACL5*** | TCACGCTCATCAACGACGAC | ACCACGTTGCGGTAAAACTC | 145 | 291 |
